# Description of the Risk Factors for Ischemic Stroke in the Lebanese Population: Their Association with Age at First Stroke Incidence and the Predictors of Recurrence

**DOI:** 10.3390/jcm14062034

**Published:** 2025-03-17

**Authors:** Jad El Masri, Diala El Masri, Maya Ghazi, Ahmad Afyouni, Hani Finge, Jad El Ahdab, Maryam Tlayss, Soltan Al Chaar, Wassim Abou-Kheir, Pascale Salameh, Hassan Hosseini

**Affiliations:** 1 INSERMU955-E01, Institut Mondor de Recherche Biomédicale, Université Paris-Est Créteil, 94000 Créteil, France; consultation.hosseini@gmail.com; 2 École Doctorale Sciences de la Vie et de la Santé, Université Paris-Est Créteil, 94010 Créteil, France; 3 Faculty of Medical Sciences, Lebanese University, Beirut 1533, Lebanon; mayanghazi99@gmail.com (M.G.); ahmadafyouni2000@gmail.com (A.A.); pascalesalameh1@hotmail.com (P.S.); 4 Department of Anatomy, Cell Biology and Physiological Sciences, Faculty of Medicine, American University of Beirut, Beirut 1107, Lebanon; wa12@aub.edu.lb; 5 Faculty of Medicine, University of Balamand, Koura 1100, Lebanon; diala.s.masri@std.balamand.edu.lb; 6 Faculty of Medical Sciences, Neuroscience Research Center (NRC), Lebanese University, Beirut 1533, Lebanon; 7 School of Medicine, Lebanese American University, Byblos 1102, Lebanon; 8 Department of Neurology, Faculty of Medical Sciences, Lebanese University, Beirut 1533, Lebanon; hafinge@hotmail.com; 9 Department of Neurology, Neurological Institute, Cleveland Clinic, Cleveland, OH 44195, USA; elahdaj@ccf.org; 10 Faculty of Arts and Sciences, University of Balamand, Koura 1100, Lebanon; maryamtlayss.tlayss@std.balamand.edu.lb; 11 Doctoral School of Science and Technology, Lebanese University, Beirut 1533, Lebanon; salchaar@bwh.harvard.edu; 12 Faculty of Pharmacy, Lebanese University, Beirut 1533, Lebanon; 13 Department of Primary Care and Population Health, University of Nicosia Medical School, 2417 Nicosia, Cyprus; 14 INSPECT-LB (Institut National de Sant e Publique, d’Épidemiologie Clinique et de Toxicologie-Liban), Beirut 1103, Lebanon; 15 RAMSAY SANTÉ, HPPE, 94500 Créteil, France

**Keywords:** risk factors, age at stroke, ischemic stroke, stroke recurrence, epidemiology, Lebanon

## Abstract

**Background**: Stroke is the third most common cause of death in Lebanon. With many preventive strategies identified, stroke remains a national burden, especially in developing countries, where risk factors and epidemiological states are understudied. This study aims to investigate the association of sociodemographic factors and health-related risk factors with age at first ischemic stroke and its recurrence in the Lebanese population. **Methods**: A retrospective study including 214 ischemic stroke cases was carried out. Sociodemographic characteristics and health-related risk factors were assessed, in addition to disability levels (modified Rankin score (mRS)), age at first ischemic stroke incidence, and number of ischemic strokes. Data were analyzed using SPSS software version 25, including descriptive, bivariate, and multivariate analyses. **Results**: This study showed that stressful factors were significantly associated with a younger age at first ischemic stroke, such as having no partner (*p* < 0.001), having employment (*p* < 0.001), and having migraines (*p* < 0.001). However, metabolic risk factors were associated with an older age of ischemic stroke, such as hypertension (*p* < 0.001) and hyperlipidemia (*p* < 0.001). Moreover, having a partner (OR: 2.136), having a family history of stroke (OR: 2.873), having hyperlipidemia (OR: 3.71), and having atrial fibrillation (OR: 2.521) were associated with ischemic stroke recurrence. **Conclusions**: Many modifiable factors are associated with age at first ischemic stroke and its recurrence. This study sheds light on the necessity of increasing knowledge and awareness of well-known risk factors in the Lebanese population. These results suggest implementing targeted preventive strategies and highlight the importance of complying with early detection and follow-up measures.

## 1. Introduction

Stroke is a clinically defined syndrome of acute, focal neurological deficit attributed to vascular injury of the central nervous system [1]. According to the Global Burden of Disease Study 2021, stroke remained the second most common cause of death and the third most common cause of disability-adjusted life years (DALYs) among non-communicable disorders globally [2]. Ischemic stroke is the most common type, making up around 90% of total cases [3]. Prevalence in Lebanon is unknown, and some related factors are understudied. In addition, recommended guidelines were not found to be uniformly applied for all patients and in all hospitals [4].

Ischemic stroke risk factors are divided into non-modifiable and modifiable [5]. Non-modifiable risk factors include increased age, African race, male gender, family history, and genetic predisposition [2,6], while modifiable risk factors include hypertension, dyslipidemia, atrial fibrillation, myocardial infarction, smoking, and others [7,8,9,10,11].

In the Middle East, the incidence and prevalence rates for all strokes ranged between 22.7 and 250 per 100,000 population per year and between 508 and 777 per 100,000 population respectively. The mean age of stroke lies within the sixth and the seventh decade, and hypertension was the most reported risk factor. The overall case-fatality rate within one month was 12–32% [12]. In Lebanon, a study conducted in 2019 showed that the cumulative mortality rates were 14.1% at 1 month and 22% at 1 year [13].

In Lebanon, a lower–middle-income country in the Eastern Mediterranean Region, data on the prevalence of stroke subtypes and their associations with risk factors are limited [14]. Despite that, WHO classified stroke as the third leading cause of death in 2021 in Lebanon (40.9 per 100,000 population) [15]. Moreover, due to low socioeconomic status and the aging population, stroke is considered a major health problem [16]. Furthermore, modifiable risk factors also have a high prevalence in the Lebanese population [17].

Several studies addressed ischemic stroke risk factors in Lebanon, yet none addressed the age at first stroke and the recurrence [18,19,20,21,22]. Therefore, identifying key factors associated with onset and recurrence can help healthcare workers optimize their preventive strategies and interventions. This study aims to investigate the association between sociodemographic factors, health-related risk factors, and the age of first ischemic stroke and its recurrence in the Lebanese population.

## 2. Methods

### 2.1. Study Design

A retrospective cohort study was carried out to assess the effect of risk factors on the age of the first ischemic stroke incidence and the number of incidents. All participants provided informed consent, which outlined the study’s objectives and potential concerns, and ensured the confidentiality of the data collected. Participation was completely voluntary, and data were gathered from patients’ medical records between February and December 2023.

### 2.2. Participants

All included cases were Lebanese individuals admitted to Sahel General Hospital or Al Rassoul Al Azam Hospital in Beirut due to an ischemic stroke. All patients admitted to the neurology department were targeted to assess eligibility to include them. However, some eligible patients were excluded due to refusal, early discharge or transfer, or missing information.

Inclusion criteria: All included patients were 18 years old or older, with confirmed ischemic stroke by computed tomography (CT) and/or magnetic resonance imaging (MRI). In addition, diagnosis was confirmed clinically in all included patients [3].

Exclusion criteria: Patients without radiological or clinical confirmation of ischemic stroke were excluded. In addition, patients with CVA other than ischemic stroke were excluded, such as transient ischemic attack or hemorrhagic stroke [23].

### 2.3. Variables and Data Source Measures

Patients’ medical records were screened for all studied factors:

- Sociodemographic characteristics: Age, gender, marital status, educational level, employment, governorate, income level, and area of residency;
- Previous health-related conditions: smoking, family history of stroke, hypertension (defined as having systolic blood pressure >130 mm Hg and/or diastolic blood pressure >80 mm Hg [24]), hyperlipidemia (defined as having elevated lipid levels [25]), deep vein thrombosis (DVT) or pulmonary embolism (PE) (defined as having blood clots in venous circulation or in pulmonary circulation [26]), atrial fibrillation (defined as having cardiac electricity disturbance [27]), migraine (defined as having a specific type of headache [28]), and myocardial infarction (MI) (defined as having a decreased blood flow to the myocardium [29]);
- Level of disability: the modified Rankin scale was used to classify patients depending on disability levels. It ranges from 0 (no disability) to 6 (death) [30].

### 2.4. Ethical Considerations

Ethical approval was obtained from the Institutional Review Board (IRB) of Sahel General Hospital (ID number: 1/2023; approval date: 10 January 2023) at the hospitals involved, assuring that this study respects confidentiality and anonymity.

### 2.5. Statistical Analysis

Data were analyzed using SPSS software version 25. Descriptive analysis was performed using frequencies and percentages for categorical variables and means and standard deviations for continuous variables. A bivariate analysis was performed to identify the association between sociodemographics and health-related risk factors with the age at first stroke and number of stroke events. A Student’s test (in symmetrical data) or a Wilcoxon test (in asymmetrical data) was used to compare means between two groups, and an ANOVA test (in symmetrical data) or Kruskal–Wallis test (in asymmetrical data) was used to compare means between more than two groups. Chi-square and Fisher exact tests were used to compare percentages between groups. A *p* < 0.05 was considered statistically significant.

A generalized linear model (GLM) was used to investigate the association (beta and 95% CI) of marital status, educational level, employment, smoking, family history, hypertension, hyperlipidemia, atrial fibrillation, and myocardial infarction with the age at first stroke incidence. A binomial logistic regression model was performed to investigate the adjusted odds ratio (ORa) with a 95% CI of marital status, educational level, employment, smoking, family history, hypertension, hyperlipidemia, atrial fibrillation, myocardial infarction, and number of stroke events. The Hosmer–Lemeshow test was non-significant, demonstrating the test’s adequacy. The decision tree model was used to identify the significant factors influencing age at first ischemic stroke and stroke recurrence. All covariates with a *p* < 0.2 in the bivariate analysis were included in the regression models. The CI was set at 95%, and a value of *p* < 0.05 was considered significant.

## 3. Results

Table 1 represents a description of the sociodemographic characteristics of the sample included. A total of 214 ischemic stroke patients were included in this study. Around half of the sample were females (102, 48.13%). In total, 119 (55.6%) had a partner, and 95 (44.4%) did not have a partner. Around half had school education (99, 46.26%), and a quarter (51, 23.83%) were not educated. A total of 152 (71.03%) were unemployed, 31 (14.48%) were employed, and another 31 (14.48%) had a freelance profession. The majority had a moderate income (129, 60.3%), and around three-fourths lived in an urban area (159, 74.3%).

Table 2 represents a description of health-related risk factors in the sample included. Almost half of the sample were smokers (126, 58.88%), and around half had a family history of stroke (105, 49.06%). The majority had hypertension (194, 90.65%), and almost three-fourths had hyperlipidemia (151, 70.56%). The majority of patients did not have DVT or PE, atrial fibrillation, migraine, or myocardial infarction.

**Figure 1:**
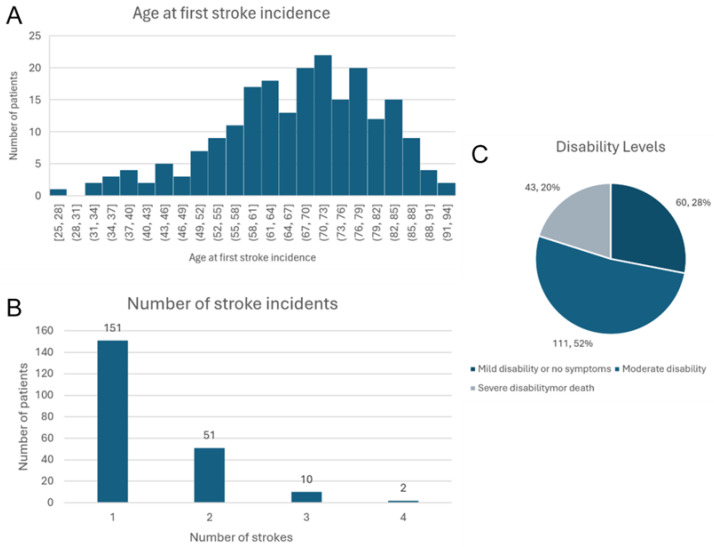
Distribution of patients by age at diagnosis (**A**), number of stroke incidents (**B**), and disability level (**C**).

Figure 1A represents the distribution of age at first stroke incidence in the included sample. The majority of patients had an ischemic stroke between the ages of 58 and 85.

Figure 1B shows the number of stroke incidents in patients, where one hundred fifty-one (70.56%) had only one stroke, fifty-one (23.83%) had two strokes, ten (4.67%) had three strokes, and only two (0.93%) had four strokes.

Figure 1C shows the distribution of patients among disability levels, where the majority (111, 52%) had moderate disability, around two-fifths had mild disability (60, 28%), and one-fifth (43, 20%) had severe disability.

Table 3 shows the association between age at first stroke and number of stroke incidents with sociodemographic factors. Being without a partner was associated with a significantly younger age at first stroke incidence (*p* < 0.001). Having no education or having only school education was associated with an older age at stroke incidence (*p* < 0.001). Similarly, having no employment was associated with an older age at first stroke incidence compared to being employed or having a freelance profession (*p* < 0.001).

As for the number of ischemic stroke events and recurrence, being uneducated and having no employment were associated with a higher number of strokes (*p* < 0.001 and *p* = 0.004, respectively) and more recurrence (*p* = 0.027 and *p* = 0.004, respectively).

**Figure 2:**
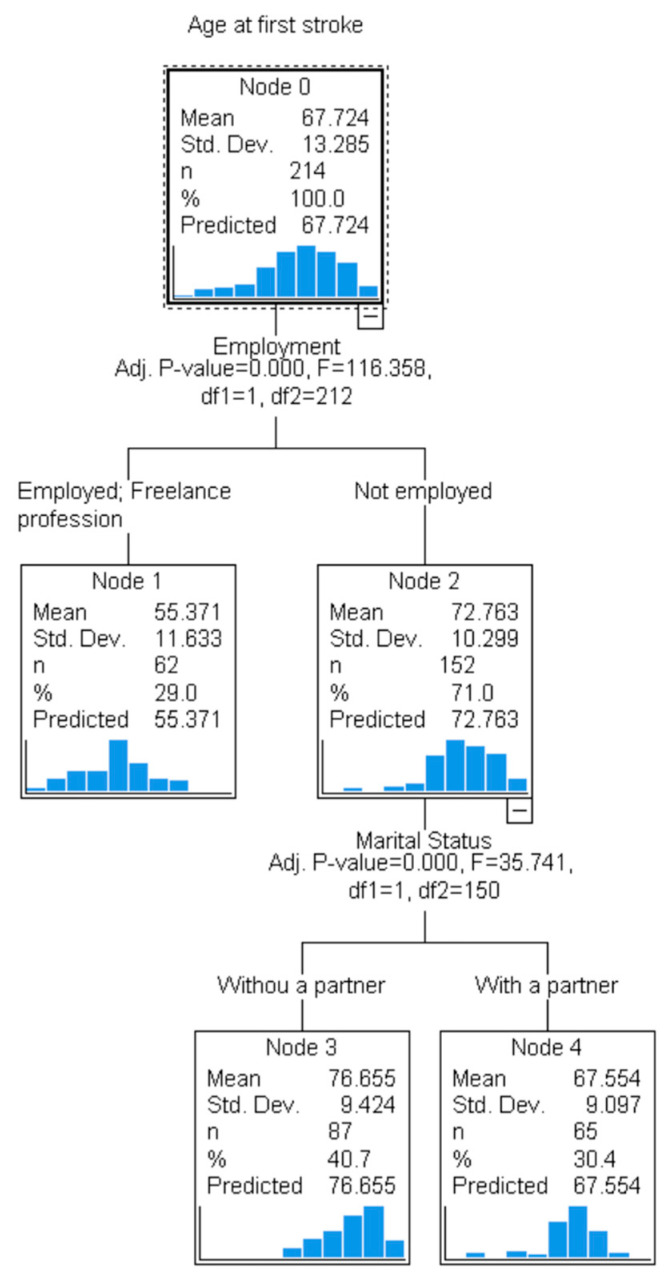
Decision tree model for age at first ischemic stroke incidence.

Figure 2 represents the decision tree model for the age at first ischemic stroke. Those not employed had a significantly older age at first stroke compared to others (*p* < 0.001). Among them, those having a partner had a significantly higher age at first stroke compared to those without a partner (*p* < 0.001).

**Figure 3:**
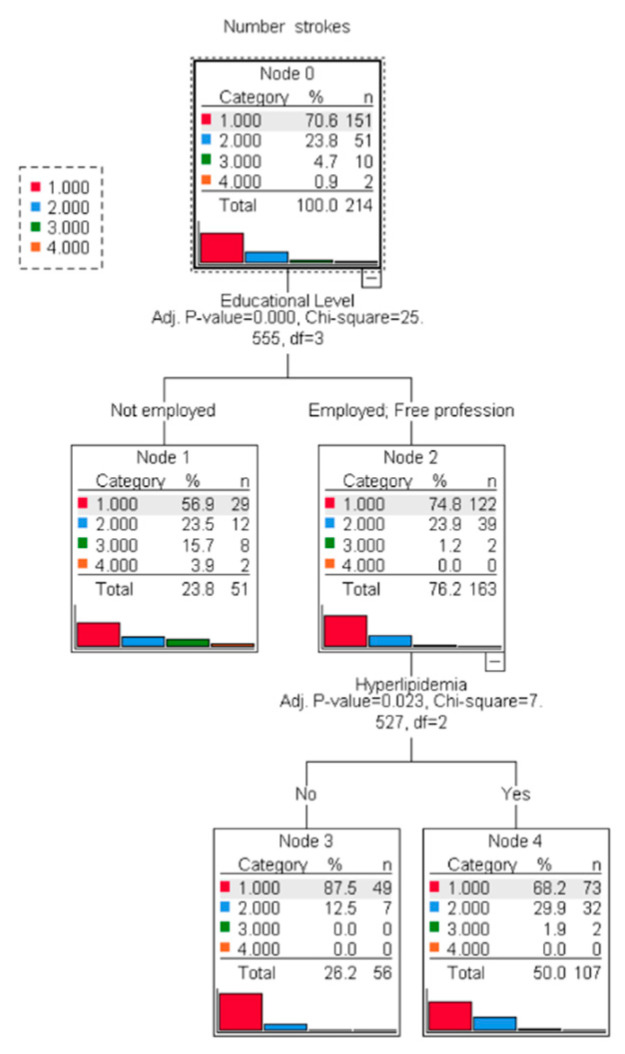
Decision tree model for number of ischemic stroke events.

Figure 3 represents the decision tree model for the number of ischemic stroke events. Those not employed had a significantly higher number of stroke events compared to other (*p* < 0.001). Among others, those having hyperlipidemia had a higher number of strokes compared to those not having hyperlipidemia (*p* = 0.023).

Table 4 shows the association between age at first stroke and number of stroke incidents with health-related risk factors. Smoking, family history of stroke, and migraine were associated with a younger age at first stroke incidence (*p* < 0.001, *p* = 0.016, and *p* < 0.001, respectively). Having hypertension, hyperlipidemia, DVT or PE, and myocardial infarction were associated with older age at first stroke incidence (*p* < 0.001, *p* < 0.001, *p* = 0.025, and *p* = 0.041, respectively). As for number of stroke incidents and recurrences, family history, hypertension, hyperlipidemia, DVT or PE, and atrial fibrillation were associated with a higher number of stroke incidents (*p* = 0.005, *p* = 0.012, *p* < 0.001, *p* = 0.041, and *p* = 0.001, respectively) and more recurrence (*p* = 0.006, *p* = 0.012, *p* = 0.001, *p* = 0.067, *p* = 0.001, respectively).

Table 5 shows the correlation between age at first stroke and number of stroke incidents with the level of disability. Younger age at first stroke was associated with milder levels of disability (*p* < 0.001), while a bigger number of stroke incidents was associated with more severe disability (*p* = 0.05).

Table 6 shows the generalized linear model taking the age at first stroke incidence with sociodemographic characteristics and health-related risk factors. Compared to having no partner, having a partner (beta = 6.058) was significantly associated with an older age at stroke incidence (*p* < 0.01). Being unemployed was significantly associated with an older age at first stroke incidence (beta = 7.98), as well as having hypertension (beta = 5.06) and hyperlipidemia (beta = 3.378). Having migraine was associated with a younger age at first stroke (beta = −9.10), as well as being a smoker (beta = −2.929).

Table 7 shows the binomial forward logistic regression regarding the recurrence of ischemic stroke (more than one incident versus one): being employed (OR: 9.591, CI 95% 1.976–46.56, *p* = 0.005) was associated with higher odds of having more than one stroke incident compared to being not employed. Similarly, having a partner (OR: 2.136, CI 95% 1.05–4.345, *p* = 0.036), having a family history of stroke (OR: 2.873, CI 95% 1.473–5.604, *p* = 0.002), having hyperlipidemia (OR: 3.71, CI 95% 1.515–9.085, *p* = 0.004), and having atrial fibrillation (OR: 2.521, CI 95% 1.272–4.999, *p* = 0.008) were associated with higher odds of having more than one stroke incidents.

## 4. Discussion

This study aimed to assess the effect of sociodemographic factors and health-related risk factors on the age at first ischemic stroke incidence and the number of ischemic stroke events. This study showed that stressful factors were significantly associated with a younger age at first ischemic stroke, such as having no partner (*p* < 0.001), having employment (*p* < 0.001), and having migraine (*p* < 0.001). However, metabolic risk factors were associated with an older age of ischemic stroke, such as hypertension (*p* < 0.001) and hyperlipidemia (*p* < 0.001). Moreover, being employed (ORa = 9.591), having no partner (OR = 2.136), having a family history of stroke (ORa = 3.251), hyperlipidemia (ORa = 3.71), and atrial fibrillation (ORa = 2.521) were associated with ischemic stroke recurrence.

Considering the effect of metabolic risk factors, Soto-Cámara R. et al. (2020) showed that hypertension was associated with an older age at first stroke event, which agrees with the results reached in this study [31]. This could be explained by the mechanistic pathway through which hypertension causes ischemic stroke. Hypertension, as well as some other diseases, such as hyperlipidemia, promotes intracranial atherosclerosis and stenoses, inducing smooth muscle hypertrophy, a reduction in the wall-to-lumen ratio, and a reduction in vessel wall compliances [32]. These processes are slow processes that are more prevalent at older ages [33]. Furthermore, it is worth mentioning that hypertensive and hyperlipidemic patients also have regular visits to physicians, which aids in the early detection and prevention of other stroke-related risk factors [25,34]. In Lebanon, where the healthcare system is suffering from multiple crises, this could offer indirect benefits to patients by offering frequent visits to healthcare individuals, which leads to better orientation in patients, and a chance to have a regular checkup [35,36]. Moreover, in several cases, antiplatelet therapy is used as secondary prevention for patients with hypertension, which prevents or at least delays the emergence of thrombotic events [37]. These facts sum up to explain the delay in stroke incidence to an older age.

As for the effect of stressful factors, several studies proved the link between stress and ischemic stroke [38,39,40]. This was suggested to result from the sudden sympathetic stimulation caused by acute stress, leading to transient ischemia and rupture of pre-formed thrombotic plaques [38]. Having no partner, which might indirectly indicate sexual activity, was associated with higher levels of stress [41]. In addition to being educated and having employment, especially in the healthcare field, this might help explain the association between younger ages and first stroke [42,43,44]. Similarly, migraine was considered a stress factor and was directly correlated to ischemic stroke [45]. As for smoking, it was found to worsen negative emotions and coping strategies, leading to elevated stress levels [46]. Furthermore, nicotine was found to increase platelet aggregation, elevate fibrinogen levels, reduce HDL cholesterol, and directly damage blood vessel walls, which all together lead to ischemic stroke [47].

Regarding the number of ischemic stroke events, both metabolic and stressful factors caused an increase in the number of strokes. Despite the fact that several stroke cases are associated with recurrence, the percentage of recurrent cases after 1 year decreased from 8% between 1995 and 1999 to 4.3% between 2015 and 2018, certainly after the development of several preventive measures [48,49]. In Lebanon, the low levels of awareness and the limited knowledge of warning signs, in addition to the poor knowledge of necessary lifestyle modifications aid against the prevention of stroke recurrence, explaining the significant recurrence rate in patients exposed to risk factors [50,51]. Similarly, social status was previously found to have a direct effect on stroke recurrence [52]. Other studies also assessed the timing of stroke recurrence, where a study on German found that the percentage of recurrent cases was 1.2% in the first 30 days, 3.4% within 90 days, and 7.4% within 1 year [53]. However, in Lebanon, no data are available on the timing of recurrence.

This study is not without its limitations. The central locations of the hospitals included may limit the generalizability of the results to all areas, as peripheral regions might have different access to healthcare facilities and exposure to risk factors. The limited sample size could reduce the power of statistical analyses, potentially leading to non-significant associations. Additionally, information bias is possible due to factors like social desirability in patient responses, recall bias—especially in severe stroke cases with cognitive impairment—and underdiagnosed conditions. Although this bias is likely non-differential and would only push results toward the null hypothesis, it remains a consideration. Furthermore, while two multivariable regression models were used to mitigate confounding, unmeasured confounders may still exist, leading to residual confounding. To address these limitations and confirm the findings, further prospective studies are recommended that take into account these weaknesses.

## 5. Conclusions

This retrospective study offers a summary of ischemic stroke age at occurrence and recurrence risk factors in Lebanon. Metabolic risk factors, including hypertension and hyperlipidemia, were associated with an older age at first ischemic stroke, while stressful factors such as having no partner, being employed, and having migraine were associated with younger ages. Moreover, being employed and having a family history of stroke, hyperlipidemia, and atrial fibrillation were associated with ischemic stroke recurrence.

Overall, these results offer a new direction to explain the mechanism through which risk factors lead to ischemic stroke, which helps in implementing targeted preventive strategies. Furthermore, this study highlights the importance of complying with early detection and follow-up measures and sheds light on the necessity of increasing knowledge and awareness of well-known risk factors.

## Figures and Tables

**Table 1:** Sociodemographic characteristics of the sample included.

Factor	Category	Number	Percentage
Age	Mean ± SD	68.589 ± 13.436	
Gender	Female	103	48.13
	Male	111	51.86
Marital status	With a partner	119	55.6
	Without a partner	95	44.4
Governorate	North	11	5.1
	Beirut	99	46.3
	South	57	26.6
	Mount Leb	20	9.3
	Bekaa	27	12.6
Education	Not educated	51	23.83
	School education	99	46.26
	University education	64	29.91
Employment	Not employed	152	71.03
	Employed	31	14.48
	Freelance profession	31	14.48
Income	Low	71	33.2
	Moderate	129	60.3
	High	28	6.5
Residency location	Rural	55	25.7
	Urban	159	74.3

**Table 2:** Description of health-related risk factors in the sample included.

Factor	Category	Number	Percentage
Smoking	Yes	126	58.88
	No	88	41.12
Family history	Yes	105	49.06
	No	109	50.94
Hypertension	Yes	194	90.65
	No	20	9.35
Hyperlipidemia	Yes	151	70.56
	No	63	29.44
DVT or PE	Yes	27	11.21
	No	187	87.39
Atrial fibrillation	Yes	74	34.58
	No	140	65.42
Migraine	Yes	28	13.08
	No	186	86.92
Myocardial infarction	Yes	49	22.9
	No	165	77.1

**Table 3:** Bivariate analysis of age at first stroke and number of strokes.

Factor	Category	Age at 1st Stroke		Number of Strokes		Stroke Recurrence		
		Mean ± SD	*p*-Value	Mean ± SD	*p*-Value	Yes	No	*p*-Value
Gender	Female	68.75 ± 14.039	0.281	1.34 ± 0.57	0.884	30 (23.07%)	73 (76.93%)	0.923
	Male	66.77 ± 12.533		1.38 ± 0.661		33 (29.73%)	78 (70.27%)	
Marital status	With a partner	74.87 ± 11.508	<0.001 *	1.37 ± 0.62	0.891	28 (29.47%)	67 (70.53%)	0.992
	Without a partner	62.02 ± 11.793		1.35 ± 0.619		35 (29.41)	84 (70.59%)	
Governorate	North	70.45 ± 12.421	0.15	1.18 ± 0.405	0.381	2 (18.18%)	9 (81.82%)	0.317
	Beirut	68.34 ± 13.313		1.39 ± 0.667		31 (31.31%)	68 (68.69%)	
	South	69.63 ± 12.059		1.28 ± 0.59		12 (21.05%)	45 (78.95%)	
	Mount Lebanon	63.95 ± 13.847		1.35 ± 0.489		7 (35%)	13 (65%)	
	Bekaa	63.11 ± 14.813		1.48 ± 0.643		11 (40.74%)	16 (59.26%)	
Education	Not educated	75.92 ± 9.718	<0.001 *	1.67 ± 0.887	<0.001 *	22 (43.14	29 (56.86%)	0.027 *
	School education	69.13 ± 10.558		1.3 ± 0.504		28 (28.28%)	71 (71.72%)	
	University education	59.02 ± 14.622		1.2 ± 0.406		13 (20.31%)	51 (79.69%)	
Employment	Not employed	72.76 ± 10.299	<0.001 *	1.44 ± 0.668	0.004 *	54 (35.53%)	98 (64.47%)	0.004 *
	Employed	51.13 ± 12.005		1.23 ± 0.425		7 (22.58%)	24 (77.42%)	
	Freelance profession	59.61 ± 9.625		1.1 ± 0.396		2 (6.45%)	29 (93.55%)	
Income	Low	67.83 ± 10.191	0.15	1.41 ± 0.667	0.767	23 (32.39%)	48 (67.61%)	0.799
	Moderate	68.02 ± 14.061		1.33 ± 0.591		36 (27.91%)	93 (72.09%)	
	High	64.5 ± 14.867		1.36 ± 0.633		4 (28.57%)	10 (71.43%)	
Residency location	Rural	66.49 ± 12.993	0.42	1.45 ± 0.689	0.241	19 (34.55%)	36 (65.45%)	0.335
	Urban	68.15 ± 13.398		1.33 ± 0.59		44 (27.67%)	115 (72.33%)	

*: Statistically significant.

**Table 4:** Bivariate association between age at first stroke and number of stroke incidents with health-related risk factors.

Factor	Category	Age at First Stroke		Number of Strokes		Stroke Recurrence		
		Mean ± SD	*p*-Value	Mean ± SD	*p*-Value	Yes	No	*p*-Value
Smoking	Yes	64.5 ± 11.43	<0.001 *	1.33 ± 0.645	0.176	32 (25.4%)	94 (74.6%)	0.121
	No	72.34 ± 14.414		1.4 ± 0.578		31 (35.23%)	57 (64.77%)	
Family history	Yes	65.5 ± 14.685	0.016 *	1.48 ± 0.681	0.005 *	40 (38.1%)	65 (61.9%)	0.006 *
	No	69.87 ± 11.442		1.25 ± 0.53		23 (21.1%)	86 (78.9%)	
Hypertension	Yes	69.42 ± 11.267	<0.001 *	1.39 ± 0.637	0.012 *	62 (31.96%)	132 (68.04%)	0.012 *
	No	51.25 ± 19.336		1.05 ± 0.224		1 (5%)	19 (95%)	
Hyperlipidemia	Yes	71.15 ± 10.471	<0.001 *	1.46 ± 0.681	<0.001 *	55 (36.42%)	96 (63.57%)	0.001 *
	No	59.51 ± 15.614		1.13 ± 0.336		8 (12.7%)	55 (87.3%)	
DVT or PE	Yes	73.07 ± 12.764	0.025 *	1.63 ± 0.839	0.041 *	51 (44.44)	136 (55.56%)	0.067
	No	66.95 ± 13.213		1.32 ± 0.571		12 (27.27%)	15 (72.73)	
Atrial fibrillation	Yes	69.95 ± 10.994	0.075	1.57 ± 0.76	0.001 *	31 (43.24%)	109 (56.76%)	0.001 *
	No	66.55 ± 14.246		1.25 ± 0.496		32 (22.14%)	42 (77.86%)	
Migraine	Yes	54.71 ± 14.616	<0.001 *	1.25 ± 0.441	0.472	56 (25%)	130 (75%)	0.58
	No	69.68 ± 11.939		1.38 ± 0.639		7 (30.1%)	21 (69.9%)	
Myocardial infarction	Yes	71.12 ± 9.482	0.041 *	1.45 ± 0.679	0.220	45 (36.73%)	120 (63.27%)	0.202
	No	66.72 ± 14.086		1.33 ± 0.598		18 (27.27)	31 (72.73)	

*: Statistically significant.

**Table 5:** Bivariate association between age at first stroke and number of stroke incidents with disability levels.

Factor	Category	Age at First Stroke		Number of Strokes		Stroke Recurrence		
		Mean ± SD	*p*-Value	Mean ± SD	*p*-Value	Yes	No	*p*-Value
mRS category	Mild disability or no symptoms	62.7 ± 15.66	<0.001 *	1.18 ± 0.39	0.05 *	11 (18.33%)	49 (81.66%)	0.082
	Moderate disability	68.43 ± 11.743		1.42 ± 0.64		38 (34.23%)	73 (65.77%)	
	Severe disability or death	72.91 ± 11.156		1.44 ± 0.765		14 (32.56%)	29 (67.44%)	

*: Statistically significant.

**Table 6:** Multivariable analysis: correlates of age at first stroke incidence with risk factors.

Parameter	B	Std. Error	*p*-Value	95% Confidence Interval	
				Lower Bound	Upper Bound
Intercept	54.753	2.733	0.000	49.365	60.141
Marital status					
With partner vs. no partner *	6.058	1.385	0.000	3.326	8.789
Education					
Not educated vs. university education *	5.512	1.828	0.003	1.906	9.117
School vs. university education *	2.246	1.561	0.152	−0.832	5.324
Employment status					
Not employed vs. freelancer *	7.983	1.896	0.000	4.244	11.721
Employed vs. freelancer *	−4.518	2.246	0.046	−8.947	−0.088
Other lifestyle factors and comorbidities					
Smoking	−2.929	1.316	0.027	−5.525	−0.334
Family history of stroke	−2.303	1.170	0.050	−4.611	0.004
Hypertension	5.064	2.256	0.026	0.615	9.513
Hyperlipidemia	3.378	1.455	0.021	0.509	6.248
DVT or PE	−0.939	1.808	0.604	−4.504	2.626
Atrial fibrillation	−1.337	1.271	0.294	−3.844	1.170
Migraine	−9.109	1.817	0.000	−12.693	−5.525
Myocardial infarction	2.477	1.434	0.086	−0.351	5.304

*: reference; dependent variable: age at first stroke.

**Table 7:** Multivariable analysis: forward logistic regression taking the recurrence of stroke incidents with sociodemographic and risk factors.

Parameter	*p*-Value	ORa [Exp(B)]	95% Confidence Interval	
			Lower	Upper
Employment status	0.018			
Employed vs. Not employed *	0.005	9.591	1.976	46.56
Freelancer vs. Not employed *	0.044	6.036	1.053	34.61
Partner vs. No partner *	0.036	2.136	1.05	4.345
Family history of stroke	0.001	3.251	1.625	6.505
Hyperlipidemia	0.004	3.710	1.515	9.085
Atrial fibrillation	0.008	2.521	1.272	4.999
Constant	0.000	0.011		

*: reference category; dependent variable: stroke recurrence.

## Data Availability

Data are available upon request from the corresponding author.
